# Human Health Risk Assessment of Informal Slaughter by Small‐Scale Farmers in Gauteng Province, South Africa, Focusing on *Brucella abortus*

**DOI:** 10.1155/vmi/9914666

**Published:** 2026-03-10

**Authors:** Gillian Declercq, Chin-Chi Liu, Anita Luise Michel

**Affiliations:** ^1^ Department of Veterinary Tropical Diseases, Faculty of Veterinary Science, University of Pretoria, Private Bag X04, Onderstepoort, Pretoria, 0110, South Africa, up.ac.za; ^2^ Department of Veterinary Clinical Sciences, School of Veterinary Medicine, Louisiana State University, Skip Bertman Drive, Baton Rouge, 70803, USA, lsu.edu; ^3^ Office of Research and Graduate Education, School of Veterinary Medicine, Louisiana State University, Skip Bertman Drive, Baton Rouge, 70803, USA, lsu.edu

**Keywords:** *Brucella*, brucellosis, food safety, Gauteng, informal slaughter, meat inspection, public health, zoonotic risk

## Abstract

**Background:**

Informal livestock slaughter is a common and legal practice in South Africa. It is performed by untrained community members permitted for cultural and religious purposes and for weddings, funerals, and subsistence. It is exempted from official meat inspection.

**Objective:**

To investigate the practice of informal livestock slaughter in small‐scale farmers in south and eastern Gauteng Province with regard to the frequency and the associated zoonotic risk factors with an emphasis on brucellosis.

**Methodology:**

During the period 2017–2018, structured interviews were conducted in one‐on‐one sessions during which a pre‐tested questionnaire was completed. The questionnaire covered demographics, livestock information, informal slaughter practices, and veterinary public health and was delivered in one of the locally spoken languages.

**Results:**

A total of 108 participants were enrolled in the study but not all questions were answered by each respondent. Informal livestock slaughter, predominantly of cattle, was commonly practiced by 64.0% of respondents at least once per year, with higher frequencies reported among younger individuals (< 36 years). In most cases (86.2%), the slaughter was performed by the farmers themselves or a family member. Cultural and religious events, weddings, or funerals were the most common purposes (59.0%), followed by home consumption (26.0%) and sale of products (9.1%). Personal protective equipment was used by 59.1% of participants overall, with the lowest usage observed among younger individuals (18–35 years; 38.5%).

Offal, including lymph nodes, is consumed regularly and mostly cooked, but occasionally raw. Some respondents reported slaughtering sick animals and consuming abnormally appearing organ parts.

**Conclusions:**

This first structured survey of informal slaughter risk factors in Gauteng identified multiple practices that pose risks for the zoonotic transmission of *Brucella* and other food‐borne pathogens associated with informal livestock slaughter. The findings highlight the need for education of livestock owners on the disease prevention and transmission as well as the development of relevant national guidelines alongside the Meat Safety Act.

## 1. Introduction

Animal slaughter has historically played an important role in many cultures and religions worldwide. Beyond its role as a source of protein, animal slaughter represents a means of sacrifice and atonement in many religious traditions [1].

In South Africa, livestock slaughter is regulated by law [2] and is performed both at formal abattoirs and informally by communities [2]. Informal slaughter, also referred to as traditional slaughter, is a frequently occurring but poorly understood practice [3–5] that is legally provisioned and supported both by the Constitution of South Africa, (Section 15(1), which allows for the expression and practice of religious and cultural beliefs (Act 108 of 1996) [6]. The Meat Safety Act (Act 40 of 2000) in its Section 7 [2] permits the slaughter of livestock outside an abattoir when the purpose is for own consumption, cultural practices, and/or religious purposes. However, informal slaughter practices are not regulated by veterinary public health authorities or food safety controls to prevent unsanitary meat handling or to ensure meat inspection [2].

Meat quality and safety can be compromised by food‐borne pathogens that enter meat through contamination during or after the slaughter process or through slaughter animals infected with diseases transmissible to humans, also referred to as zoonoses [7]. Examples of zoonotic and food‐borne illnesses that may be transmitted from uncontrolled slaughter due to a lack of formal meat inspection include salmonellosis, anthrax, Rift Valley fever, toxoplasmosis, brucellosis (BR), taeniasis, cysticercosis, zoonotic tuberculosis, campylobacteriosis, and *Escherichia coli* infections [3, 7, 8].

In South Africa, livestock diseases with significant impacts on human health and the economy are controlled under the Animal Diseases Act [9]. For major diseases, including bovine brucellosis (bBR) and bovine tuberculosis (bTB), the state has implemented specific disease control programs aimed at safeguarding the public from their impacts. After near‐eradication in the 1980s, a slow re‐emergence of bTB has been observed in the country [10, 11]. Similarly, the BR control program in cattle showed initial successes in the mid‐1980s [12]; however, the disease is currently poorly controlled, with rising seroprevalence [13]. In the Gauteng Province (GP), a recent serosurvey revealed a herd prevalence of 13.7% [14], which had remained unchanged from the previous assessment conducted four years earlier [15].

Human exposure to BR is known to occur through contact with infected livestock during obstetrical manipulations, contact with aborted material, or slaughtering and flaying of the carcass of infected animals [12, 16]. The burden of human BR in Africa, including South Africa, remains unknown as national incidence rates have not been updated for decades, despite sporadic human cases being reported [17]. Human exposure during the slaughter process occurs through mucous membranes and breaks in the skin via contact with infected tissues and inhalation of aerosolized body fluids [14, 18]. Measures to limit disease exposure during slaughter include the use of personal protective equipment (PPE) such as overalls, gumboots, gloves, and masks, typically implemented in specifically prepared abattoirs by trained personnel [19, 20].

The lack of meat safety and hygiene standards in informal slaughter significantly increases the risk of transmission of zoonotic pathogens including food‐borne infections. This elevated risk endangers both consumers and individuals involved in the preparation of meat and viscera from informal slaughter [21].

This study aimed to describe zoonotic risk factors associated with informal slaughter practices among small‐scale farmers in the south and eastern regions of Gauteng Province, with a particular emphasis on BR.

## 2. Materials and Methods

### 2.1. Study Area

Gauteng is the social melting pot of South Africa, presenting the greatest cultural and religious diversity and the highest population density in the country [22]. This study was carried out in the southern and eastern parts of Gauteng, regionally classified as the Germiston State Veterinary area for Gauteng Veterinary Services (GVS) during 2017–2018.

### 2.2. Study Participants

Study participants were small‐scale farmers, defined as livestock owners who use animals for subsistence purposes or small‐scale production without generating profit. These farmers utilized either communal grazing land or private plots of land smaller than 25 Ha [23].

Participant enrollment was based on convenience sampling during routine primary animal healthcare visits provided by veterinary officials from the GVS, a division of the Gauteng Department of Agriculture and Rural Development (GDARD). Farmers receiving GVS assistance were those with limited or no access to private veterinary services and relied on state veterinary assistance for livestock health and management, as well as disease control and prevention. All small‐scale farmers requesting and receiving veterinary services during the enrollment period were invited to participate in the study. Participation was voluntary and not linked to the provision of veterinary services.

Farmers who consented in writing to participate in the study were enrolled by a GVS official (interviewer) who had received prior training in conducting research interviews. In cases where participants were illiterate, the consent form was explained verbally. If the participant was unable to sign, the interviewer signed as a proxy, and the participant marked an “X” on the form to indicate consent. The interviewer explained the purpose of the study and informed participants that participation was voluntary and that they could withdraw at any point during interview.

A pilot study was performed with ten small‐scale farmers using the same questionnaire interview in the method planned for the full study. The pilot study assessed the user‐friendliness and clarity of the questions. Based on feedback, the following modifications were made.

### 2.3. Questions Adjusted Through the Pilot Study

• Financial status: the addition of a “self‐employed” option was added as some small‐scale farmers received income from personal enterprises and were not necessarily either employed, unemployed, or on social grant.
• Frequency of working with livestock: “more than once a week” was replaced with “daily or almost daily”.
• Slaughter of injured or sick animals: addition of “for consumption” to added questions.

The structured interviews were conducted face‐to‐face using an interview guide, hereafter referred to as a questionnaire. One‐on‐one data collection sessions were scheduled at the participant’s homestead to ensure privacy. The questionnaire was provided in English and completed by the interviewer on behalf of the interviewee. The interviewers were fluent in languages commonly spoken in the study area and presented the questions in the language most convenient for both parties. For effective communication and understanding, laminated color images (A4) of target organs, lymph nodes and common organ abnormalities, were used.

The questionnaire consisted of four sections containing a total of 22 questions, some with subquestions, covering participant sociodemographics (*n* = 3), livestock information (*n* = 7), informal slaughter practices (*n* = 7), and veterinary public health knowledge (*n* = 5). The actual number of respondents answering to a particular question was reported for each section and served as the basis for calculating the response rates.

### 2.4. Data Analysis

Data were transferred from the completed questionnaires and captured into a Microsoft Excel spreadsheet and structured in a tabular format, with each respondent assigned to a single row and each question represented in a separate column.

All statistical analyses were performed using JMP Student Edition 18.2.0 (JMP Statistical Discovery LLC, Cary, NC). Chi‐square analysis was used to examine associations between categorical variables. Ordinal logistic regression was applied to identify factors influencing risky behavior associated with informal slaughter and consumption practices, including age versus slaughter frequency and PPE usage versus slaughter frequency. Binary logistic regression was used to estimate odds ratios of PPE use across age groups and to evaluate associations among age, PPE, and zoonotic disease knowledge. Statistical significance was set at *p* < 0.05.

### 2.5. Ethical Considerations

This study received ethical approval from the Department of Humanities (reference no. 28024525/GW20170813HS), Department of Health Sciences (reference no. GW20170813HS), and Animal Ethics (reference no. V100‐17) Committees of the University of Pretoria.

## 3. Results

A total of 108 participants were enrolled in the study; however, not all participants responded to every question. The number of respondents is provided for each finding in the body of the text and/or in Table 1.

**TABLE 1:** Selected data relating to informal slaughter captured from 108 small‐scale farmer participants.

	Category	Frequency	Percentage
Respondent age (*n* = 93)	18–35 yrs	18	19.4
	36–55 yrs	41	44.1
	> 55 yrs	34	36.6

Household size (*n* = 82)	1	5	6.1
	2	10	12.2
	3	11	13.4
	4	13	15.9
	5	11	13.4
	6 and more members	32	39.0

Financial status (*n* = 93)	Currently employed	17	18.3
	Unemployed	29	31.2
	Self‐employed	15	16.1
	SASSA grant recipient	12	12.9
	Pensioner	20	21.5

Livestock ownership (*n* = 93)	Yes	81	87.1
	No	12	12.9

Livestock owned (*n* = 80; more than one answer possible)	Cattle	67	86.1
	Sheep	36	45.6
	Goats	41	51.9
	Pigs	20	25.3
	Horses/donkeys	2	2.5
	Others	9	11.4

Livestock handling (*n* = 80)	Daily	56	70.0
	Weekly	10	12.5
	Monthly	14	17.5
	Less than monthly	0	0.0

BR and bTB testing of cattle (*n* = 77)	Tested for BR	44	57.1
	Positive for BR	2	4.7
	Never tested for BR	27	35.1
	Tested for bTB	37	48.1
	Positive for bTB	0	0.0
	Never tested for bTB	35	45.5
	Unsure of testing	11	14.2

Bovine abortions (*n* = 86)	Yes	22	25.6
	Size (*n* = 19)		86.4
	Rat	1	5.3
	Cat	3	15.8
	Dog (Africanis)	7	36.8
	Full‐term calf	8	42.1
	No	57	66.3
	Do not know	7	8.1

Purpose of informal slaughter (*n* = 77)	Home consumption	20	26.0
	Cultural/religious	35	45.5
	Wedding/funeral	15	19.5
	Sale of meat	7	9.1

Slaughter frequency (*n* = 89)	Once/twice per week	0	0.0
	Once/twice per month	13	14.6
	Once/twice per year	44	49.4
	Never	32	36.0

Source of slaughter animals (*n* = 75; more than one answer possible)	Own animals		
	Yes	44	58.7
	No	31	41.3
	Community	23	52.3
	Auctions	17	38.6
	Roadside sale	4	9.1

PPE (*n* = 66)	Yes	39	59.1
	No	27	40.9

PPE per age group (*n* = 66)	18–35 yrs (*n* = 13)	5	38.5
	36–55 yrs (*n* = 29)	16	55.2
	> 55 yrs (*n* = 24)	18	75.0

Offal consumption (*n* = 71)	Yes	67	94.4
	No	4	5.6

Lymph node recognition and consumption (*n* = 68)	Recognition	43	63.2
	Consumption	12	17.6
	No consumption	31	45.5
	No recognition (with consumption)	25	36.8

Lymph node consumption (*n* = 68)	Yes^∗^	37	54.4
	No	31	45.6

Preferred method of disposal for unused parts (*n* = 150 for 3 variables)		a b c Σ(a‐c)	
	Feed to dogs	3 16 21 40	26.7
	Burn in fire	1 13 15 29	19.3
	Bury	13 24 33 70	46.7
	Discard in veld	3 7 1 11	7.3

Zoonotic awareness (*n* = 91)	Yes	75	82.4
	No	16	17.6

Source of zoonosis (*n* = 71; more than one answer possible)	Live animal	49	69.0
	Dead animal	44	62.0
	Slaughtered animal	17	23.9

*Note:* SASSA: South African Social Security Agency; PPE: personal protective equipment; a: abortus, b: abnormal carcass parts; c: carcass leftover.

^∗^Included respondents who did not recognize lymph nodes.

### 3.1. Sociodemographic Information

The age distribution, household size, and employment status are summarized in Table 1. Overall, participants who were not earning an income constituted 65.6% of the study population. Study participants revealed *predominantly* gregarious living arrangements, with the majority (81.7%) residing in households of three or more individuals, compared to those living alone (6.1%) or in pairs (12.2%) (Table 1).

### 3.2. Livestock Information

The majority *of* respondents (87.1%) owned livestock, including all participants older than 55 years (*n* = 34), while the remaining 12.9% were livestock handlers or farm managers*.* Livestock ownership patterns showed that cattle (86.1%), goats (51.9%), and sheep (45.6%) were the most commonly owned species *among* small‐scale farmers. The frequencies of animal handling ranged from daily (70.0%) to at least monthly (17.5%).

With regard to mandatory disease surveillance for brucellosis (BR) and bTB, caused by *Mycobacterium bovis* (*M. bovis*), 77 respondents provided information. Of these, 57.1% and 48.1% reported that their cattle herds had previously been tested for BR and bTB, respectively, while 35.1% and 45.5% reported that their herds had never been tested. The remaining respondents (7.8% for BR and 6.6% for bTB) were unsure of the testing status. Two herds were reportedly positive for BR with no known follow‐up interventions.

A quarter of the 86 respondents (25.6%) reported abortions in their cattle herds during the six months preceding the study. A considerable proportion of the aborted fetuses (42.1%) were described as near the size of a full‐term calf and covered with hair, while smaller fetuses were most commonly described as comparable in size to a medium‐sized dog (36.8%). When asked about the disposal method for the abortus, 15.0% of respondents reported feeding aborted material to their dogs (Table 1).

### 3.3. Informal Slaughter

The purpose and frequency of informal slaughter events in the study area were determined among 77 and 89 respondents, respectively (Table 1). The most commonly reported motivation for informal slaughter was for cultural or religious purposes (45.5%), followed by home consumption (25.9%), special occasions such as weddings and funerals (19.5%), and sale of the slaughter products (9.1%).

The highest frequency of slaughter activity (once or twice per month) was observed predominantly in the 18–35 year age group (14.6%). This youngest age group (18–25 years) had significantly higher odds of engaging in more frequent slaughter activities compared to all older age groups: 18.5 times higher than those older than 56 years (95% CI: 3.1–110.2, *p* = 0.0014), 23.4 times higher than those aged 26–35 years (95% CI: 3.0–182.9, *p* = 0.0027), and 15.9 times higher than those aged 36–55 years (95% CI: 2.7–92.6, *p* = 0.0020). No other pairwise age group comparisons demonstrated statistically significant differences in slaughter frequency (all *p* > 0.05).

Across all age groups, the majority of respondents (49.4%) reported engaging in informal slaughter once or twice a year, while 36.0% reported never engaging in informal slaughter, with similar proportions across age categories.

Most of the 65 respondents (47.7%) reported preferring to slaughter livestock themselves or to have a family member perform the slaughter, irrespective of age group. Alternatively, respondents reported approaching a friend (7.7%) or hiring a slaughterer (13.9%). All but one participant indicated that the slaughterer was male.

Among the 63 respondents, the preferred livestock species for informal slaughter were cattle (60.3%), followed by goats (25.4%), sheep (22.2%), pigs (19.1%), and poultry (9.5%). Donkeys and horses were reportedly not slaughtered by participants. Regarding the sex of animals selected for slaughter, 44.1% of the 59 respondents indicated no preference, while 28.8% and 13.6% preferred male and female animals, respectively. Most of the 75 respondents (58.7%) reported a preference for slaughtering their own animals, while alternative sources for purchasing animals included farmers within the community (52.3%), livestock auctions (38.6%), and roadside sales (9.1%)

### 3.4. Veterinary Public Health

The slaughter of physically injured animals (e.g., with fractures and wounds) for consumption purposes was confirmed by 50 of the 76 respondents (65.8%). In the case of any animals showing signs of illness, 13.3% of respondents indicated that they would slaughter the animal and consume the products, while the remaining respondents rejected this option.

The consumption of offal was a common practice for 94.4% of the 71 respondents. The most commonly consumed organs were the liver (86.6%), intestines (67.2%), heart (62.7%), lungs (56.7%), and rumen (52.2%). Kidneys (44.8%), diaphragm (41.8%), and reproductive organs, specifically the uterus (3.0%), were consumed less frequently. While 78.8% of respondents commonly prepared the offal by prolonged cooking (> 30 min), the liver, kidney, and heart were generally cooked for shorter periods of 10–30 min. One respondent indicated that under certain circumstances, specified organs, namely, the liver, heart, and small sections of the rumen were consumed raw.

When a damaged or diseased organ was encountered during slaughter, 31 of the 66 respondents (47.0%) reported discarding the entire affected organ, while 19 respondents (28.8%) avoided the use of any of the organs from an affected animal. Removal of only the affected portion was selected by 15 farmers (22.7%). A single respondent confirmed consumption of an organ despite the presence of visible lesions or damage. If diseased or damaged organs or organ sections were removed, they were disposed of by either burial, feeding them to their dogs, burning them in fire, or discarding them in the veld.

When questioned about the recognition of lymph nodes in the slaughtered animal, 36.8% of the 68 respondents revealed a lack of knowledge of lymph nodes as anatomical structures. The inability to recognize lymph nodes persisted further explanations and the use of visual aids. Among respondents who were able to recognize lymph nodes, 17.6% reported consuming them together with surrounding tissue structures, resulting in a total of 63.1% of respondents consuming lymph nodes (Table 1). All participants who acknowledged lymph node consumption stated that they were consumed cooked and never raw.

The most commonly identified carcass parts that remained unused after slaughter were specified as the horns (30.3%) and the reproductive organs (27.3%), while other parts including bones, hooves, skin, tail, and feet were discarded less frequently (< 10.0%). These parts were either buried (37.5%), fed to the dogs (25.0%), burnt in fire (20.3%), or discarded in the veld (10.9%) or by undisclosed methods (6.3%).

Assessment of the frequency of use and adequacy of PPE during the informal slaughter showed that 59.1% of the 66 respondents used overalls (85.3%) and/or gumboots (67.7%). Fewer respondents reported using gloves (35.3%), mask (8.6%), or eye protection (2.9%). Among PPE users, the most common combination was overalls and gumboots (41.0%), followed by gumboots and gloves (15.0%), overalls and gloves (6.0%), or gumboots and a mask (6.0%). No respondents reported wearing a full complement of PPE considered adequate to prevent disease transmission.

Across age groups, PPE use and adequacy increased with age (Table 1). The highest frequency of PPE use was observed in respondents older than 55 years (75.0%), which was significantly higher than the 38.5% usage reported among those aged 18–35 years old (*p* < 0.05).

Participants older than 56 years had 18.0 times higher odds of using PPE compared to those aged 18–25 years (OR = 15.0, 95% CI: 1.9–319.9, *p* = 0.0080). This represented the strongest association, indicating that the oldest age group is substantially more likely to use protective equipment during slaughter activities compared to the youngest group.

This study also assessed the knowledge of zoonotic diseases among small‐scale farmers in the study area. Among the 91 respondents, the majority (82.4%) indicated awareness that diseases can be transmitted from animals to humans. Many respondents acknowledged that zoonotic diseases could originate from dead animals (62.0%) and live animals (69.0%), while only 23.9% considered zoonotic disease transmission through slaughter a possibility. Binary logistic regression revealed that age was not a significant predictor of zoonotic knowledge (*p* = 0.082). However, the small sample size in the youngest age group may have limited the interpretation of these findings. Interestingly, while zoonotic knowledge was not a statistically significant predictor of PPE usage (*p* = 0.103), participants with zoonotic knowledge showed a higher proportion of PPE usage compared to those without such knowledge. The *p* value approaching 0.05 therefore suggested a possible trend that warrants further investigation with larger sample sizes.

**FIGURE 1:**
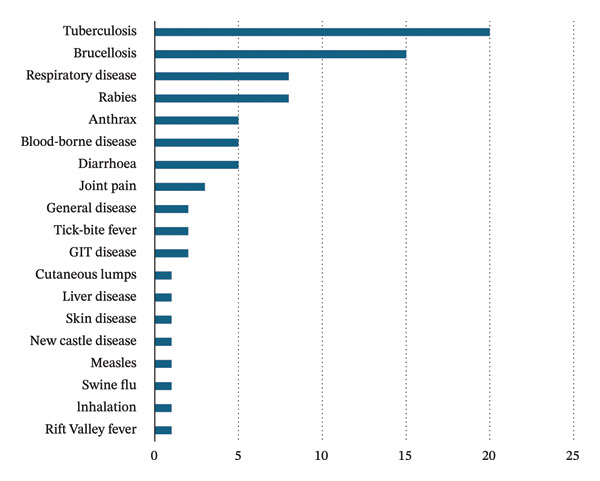
Diseases/syndromes identified by respondents as transmissible from animals to humans.

When asked to elaborate on specific diseases that could be transmitted, those most mentioned were tuberculosis and BR (Figure 1).

## 4. Discussion

A questionnaire survey with face‐to‐face interviews was conducted among 108 small‐scale farmers in the southern and eastern regions of GP. The aim was to qualitatively assess whether informal livestock slaughter and related practices posed a potential risk of zoonotic pathogen transmission, with a particular focus on BR. The findings revealed the regular practice of informal slaughter of mostly cattle for cultural or religious purposes (45.5%) most often done annually. The majority of respondents were 36–55 years of age followed closely by those over 55 years of age (Table 1). The significant ownership of livestock among these age groups, combined with the lack of income, suggests that raising livestock may not only serve as a retirement plan but also secures livelihoods and provides families with a vital source of animal‐based protein.

The collected data confirmed that slaughter‐related practices corroborated both the direct and indirect risks of zoonotic exposure during and after the slaughter process. The informally slaughtered animal carcass is not routinely subjected to meat inspection [24] and bears a zoonotic infection risk through direct exposure during animal handling and performing the informal slaughter, as well as indirect exposure through consumption.

The small‐scale farmers interviewed indicated that they slaughtered both their own and purchased animals, sometimes including sick and injured animals. It is noteworthy that sick animals encompass a higher health risk, whether the cause of illness is infectious or noninfectious. The purchase of animals for slaughter, whether from other farmers or auctions, increases disease risk, given the fact that BR had been identified in the study area [25, 26] and some study participants reported that their cattle had tested positive for BR. The actual BR occurrence might be higher since 35.1% of respondents reported that their herds had never been tested for BR. Livestock auctions, where livestock from many different owners gather, probably present the most notable risk of encountering infected animals, as the movement permit system and the BR and bTB health certificates intended to ensure that only livestock free of these diseases are traded, are poorly enforced [27, 28].

The majority of small‐scale farmers in this study (70.0%) personally handled livestock on a daily basis. This frequency and closeness of contact make livestock farmers an occupational high‐risk group for zoonoses [29]. Farmer exposure to *Brucella* organisms, in particular, occurs via contact with aborted fetuses, placenta, and abortion fluids [14]. In this study, 25.6% of respondents had experienced abortions in their cattle during the preceding six months. Although these could be due to various infectious and noninfectious causes, the described appearance of the abortus in many of these cases reflected the characteristics of an abortus of six to nine months gestational age which is considered pathognomonic for BR [16, 19].

BR is also one of the major occupational zoonoses potentially transmitted during livestock slaughter [30–32] via mucous membranes, open wounds, or the respiratory tract. A study by Kolo et al. (2024) found that 29.3% of abattoir workers in three low‐throughput abattoirs in GP had serum antibodies against *Brucella*, which is likely the result of exposure to BR‐infected animals during slaughter [13]. These data emphasize the zoonotic risk to the study population that performs informal slaughter of livestock in the study area and beyond.

One of the important pillars in the prevention of zoonotic infections during formal slaughter is the use of PPE recommended by the South African Department of Agriculture, Forestry and Fisheries [20]. These include white gumboots, jackets, pants, aprons, gloves, and masks. However, no guidelines on PPE and hygiene best practices are available for informal slaughter. This study revealed that only 59.1% of the respondents involved in informal slaughter used some form of PPE. The PPE comprised mainly overalls (85.3%) and/or gumboots (67.7%) and was considered inadequate to protect from zoonotic pathogens that spread through aerosol, such as *Brucella* [18]. This finding closely reflected the 62.6% PPE usage observed by Qekwana et al. (2017) among informal slaughterers of goats in GP [24].

In our study, PPE usage was significantly more prevalent in those older than 55 years (75.0%) compared to those in the younger generation of 18–35 years old (38.5%). A study in Nigeria among meat handlers at abattoirs found older workers to be more knowledgeable, experienced, and compliant with applying PPE and personal hygiene measures despite inadequate training [33]. Although we cannot extrapolate these findings to our study population, we speculate that older small‐scale farmers may have wisdom that comes with age or previously taught patterns and handed down safe handling routines associated with informal slaughter and meat handling. On the contrary, our findings suggested that the younger respondents who presumably lacked the experience of the elders, showed far less acceptance of the use of PPE and may underestimate the risks. This has a knock‐on effect as nearly 30% of this age group reportedly slaughtered livestock themselves once or twice a month. This frequency is higher than needed to satisfy home consumption and may indicate that they offered their workforce as slaughterers. Taken together and adding the fact that small‐scale farmers had no access to training in hygienic meat handling, this group constituted the highest risk group among the participants not only for contracting but also for disseminating zoonotic pathogens through risky informal slaughter practices. Cleaning and disinfecting hands and equipment, avoiding contact of contaminated skin with internal organs, and preventing contact between abnormal organs and the carcass are other essential hygiene practices highly relevant to limit disease transmission and contamination of the carcass during flaying and evisceration, ultimately leading to a redistribution of bacteria present on the animal’s skin and in the intestines [34, 35]. Qekwana observed in a study on informal slaughter of goats in South Africa that neither goat owners nor informal goat vendors associated food‐borne diseases with poor slaughter hygiene [36].

Indirect exposure to zoonotic pathogens is mediated by contaminated animal products and affects consumers of those products. A high proportion of study participants (81.7%) lived in multiperson households of three or more members (Table 1). As communalistic living of different age groups increases the transmission and impact of infectious diseases [37], the informal slaughter of infected livestock and/or the unhygienic handling of meat can cause the spread of zoonotic pathogens and food‐borne illnesses at the household level. This impact is disproportionally amplified at the level of cultural and religious events or weddings and funerals as they may be attended by hundreds of people.

Concerning the risk of food‐borne zoonoses, several study findings are of significance to public health. The vast majority of respondents (94.4%) consumed offal in addition to meat (Table 1). The heart, liver, lungs, intestines, and kidneys were the preferred organs, commonly subjected to cooking for at least 30 min by most. Although this treatment can be considered effective in eliminating viable *Brucella* organisms, *M*. *bovis*, and many other disease‐causing agents [38, 39], the same does not apply to shorter holding times since the minimum internal temperature to destroy pathogens may not be reached [40]. The consumption of raw heart, liver, and blood (bobete (Phalafala n.d.)) exposes consumers to a high food safety risk, especially if they were derived from sick animals or poorly bled carcasses. This is because major food pathogens, including *Brucella*, *M. bovis*, *Salmonella*, and *E. coli,* are known to spread haematogenously throughout the body and to blood‐rich organs during the bacteraemic stage of infection. They can subsequently accumulate in the associated lymph nodes, where they can remain viable for longer periods and be detected at slaughter in healthy‐appearing animals [14, 16, 41–43]. In addition, meat obtained through informal slaughter is often consumed immediately after slaughter, when the meat pH is high, which supports the survival of the aforementioned food pathogens [44].

The lack of official meat inspection incision of the lymph nodes of the head and the major organs removes this critical control point from the informal slaughter process [45]. In this study, 36.8% of respondents did not recognize lymph nodes as separate anatomical structures, while 17.7% recognized them but consumed the lymph nodes anyway. In light of the high level of offal consumption, this reinforces the food safety risk mentioned earlier. More specifically, *Brucella* spp. and *M. bovis* often persist in high concentrations in the lymph nodes of an infected animal [16, 46]. In the case of bTB, lymph nodes can contain or be entirely replaced by granulomatous lesions containing very high amounts of viable bacteria [47].

When tested, knowledge of zoonotic diseases showed similar results across various age groups, indicating a high awareness level of 82.4%. bTB and BR were most frequently identified as zoonoses, possibly due to regular extension activities by GVS. On the subject of how people could contract zoonoses, live and dead animals were mentioned at 69.0% and 61.0%, respectively, but fewer respondents (23.9%) believed that zoonotic transmission could occur during livestock slaughter. This may partially explain the low uptake of PPE and presents an important opportunity for extension services to include the public health risks of informal slaughter in their education and awareness programs for farmers.

It was a concerning observation in this study that aborted fetuses and abnormal organ or carcass parts considered unfit for human consumption were commonly fed to dogs as a way of disposal. This practice can lead to infectious diseases in these animals and potentially further dissemination of the organisms back to humans or livestock [48, 49].

Lastly, we believe that survey errors and their impact in surveys carried out by face‐to‐face interviews are largely underestimated by researchers. A total of 108 small‐scale farmers, representative of the target population, were interviewed in our study, but not all parts of the questionnaire were answered by each respondent. The underlying factors for the high nonresponse rate were unknown but are not unique to this study. On the contrary, the basic assumptions most researchers make, that survey participants will not only be willing to respond to all questions but also to respond honestly, have been shown not to be true and can lead to survey errors and unreliable results [50].

Generally, unwillingness to share certain information is observed if questions are perceived to be inappropriate, controversial, sensitive, or could lead to embarrassment [51]. Sensitive questions may raise the concern that responses could be disapproved and potentially lead to repercussions [51], especially in face‐to‐face interviews. In our study, questions on the practice of informal slaughter and the use of PPE, the disposal of abnormal carcass parts, and others could have been perceived as sensitive and therefore answered by fewer participants, especially since the interviewers were government officials and frequently visiting the community. The same could have been true for other sensitive questions, eliciting doubt on the legality of their practices, e.g., practicing informal slaughter for the purpose of selling meat (not permitted under the Meat Safety Act).

The use of a convenience sample is a potential limitation in surveys, including this study. Although we could not enroll all small‐scale farmers entitled to receive services from GVS in the study area, we believe that the sample is highly representative of extremely resource‐constrained small‐scale farmers, who are generally excluded from access to private veterinary services.

Currently no regulations/guidelines for informal slaughter exist within the Meat Safety Act because this practice is only legal provided it is destined for own consumption or for cultural or religious purposes. Nevertheless, it is critically important to mitigate public health risks in the form of awareness campaigns to improve the knowledge of farmers and those performing informal slaughter. Basic knowledge on the prevailing zoonotic diseases, their transmission, and affordable practices to prevent them (including personal hygiene measures and PPE) goes a long way in empowering communities. It is the mandate of GVS to provide such training through their extension services. Future research can support this process on the uptake and impact of such training.

## 5. Conclusion

BR is known to be present in communal livestock herds in Gauteng Province (GP). This study revealed the poor implementation of personal protective measures against zoonotic pathogens during informal livestock slaughter. Regular consumption of high‐risk offal and associated lymph nodes poses a substantial risk of disease exposure to consumers, particularly when informal slaughter is conducted for large‐scale cultural, religious, and social events. The findings highlight the need for the development and effective communication of national guidelines, outside but aligned with the Meat Safety Act, to support the prevention of zoonotic disease transmission during informal livestock slaughter.

## Funding

This work was supported by the Belgian Directorate‐General for Development Cooperation Framework Agreement (FA4 DGD‐ITM 2017–2021).

## Conflicts of Interest

The authors declare no conflicts of interest.

## Data Availability

The data that support the findings of this study are available from the corresponding author upon reasonable request.
